# How Toll-like receptors influence Parkinson’s disease in the microbiome–gut–brain axis

**DOI:** 10.3389/fimmu.2023.1154626

**Published:** 2023-05-03

**Authors:** Ziyi Zhang, Zhihui Liu, Ao Lv, Chenhui Fan

**Affiliations:** ^1^ Department of Anesthesiology, Baotou Central Hospital, Baotou, China; ^2^ Baotou Clinical Medical College, Inner Mongolia Medical University, Baotou, China; ^3^ The First Clinical College, Shanxi Medical University, Taiyuan, Shanxi, China; ^4^ Safety Engineering, People’s Public Security University of China, Beijing, China

**Keywords:** microbiome-gut-brain axis, Parkinson’s disease, Toll-like receptors, immunity, α-synuclein, TLR2, TLR4, gut microbiome

## Abstract

Recently, a large number of experimenters have found that the pathogenesis of Parkinson’s disease may be related to the gut microbiome and proposed the microbiome–gut–brain axis. Studies have shown that Toll-like receptors, especially Toll-like receptor 2 (TLR2) and Toll-like receptor 4 (TLR4), are key mediators of gut homeostasis. In addition to their established role in innate immunity throughout the body, research is increasingly showing that the Toll-like receptor 2 and Toll-like receptor 4 signaling pathways shape the development and function of the gut and enteric nervous system. Notably, Toll-like receptor 2 and Toll-like receptor 4 are dysregulated in Parkinson’s disease patients and may therefore be identified as the core of early gut dysfunction in Parkinson’s disease. To better understand the contribution of Toll-like receptor 2 and Toll-like receptor 4 dysfunction in the gut to early α-synuclein aggregation, we discussed the structural function of Toll-like receptor 2 and Toll-like receptor 4 and signal transduction of Toll-like receptor 2 and Toll-like receptor 4 in Parkinson’s disease by reviewing clinical, animal models, and *in vitro* studies. We also present a conceptual model of the pathogenesis of Parkinson’s disease, in which microbial dysbiosis alters the gut barrier as well as the Toll-like receptor 2 and Toll-like receptor 4 signaling pathways, ultimately leading to a positive feedback loop for chronic gut dysfunction, promoting α-synuclein aggregation in the gut and vagus nerve.

## 1 Introduction

As early as 2,000 years ago, the ancient Greek philosopher Hippocrates, known as the father of modern medicine, proposed that “the source of all diseases begins in the guts”. The gut microbiome is dynamic and complex. The early gut microbiome is acquired from the mother’s vagina, skin, and surrounding environment at birth. Each person’s gut microbiome is stable and unique and is considered an individual microbial fingerprint or gut type (1). With the development of medicine, many studies have shown that the gut microbiome may affect the body’s brain structure and be related to neurological lesions through a special “gut–brain axis”. The microbiome–gut–brain axis links the enteric nervous system and the central nervous system with the neuroendocrine–immune system, forming a two-way signaling pathway, and the gut microbiome can participate in the regulation of the nervous system through the release of neurotransmitters, endocrine, immunity, and other pathways. As the body’s largest immune system, the immune system of the gut is essential for the protection of the body. Parkinson’s disease, the second most affected neurodegenerative disease in the world, interestingly, is thought to be inextricably linked to the role of the gut microbiome. The pattern recognition receptor Toll-like receptors, especially Toll-like receptor 2 (TLR2) and Toll-like receptor 4 (TLR4), expressed in immune cells can recognize various exogenous and endogenous molecules to mediate the production of neuroinflammation; specifically, targeting Toll-like receptors may be an important way to regulate neuroinflammation and treat diseases. Parkinson’s disease is a common neurodegenerative disease with 7 million to 10 million Parkinson’s disease patients worldwide (2), which is the “third killer” of health and life in middle-aged and elderly people after tumors and cardiovascular and cerebrovascular diseases (3). It is characterized by abnormal aggregation of α-synuclein (α-syn) in neuronal cytoplasm to form Lewy bodies (LBs) and degeneration necrosis of dopaminergic neurons in the substantia nigra of the midbrain (4). In turn, α-synuclein aggregates may migrate to the brain via peripheral nerves such as the vagus nerve, leading to nerve damage and neurodegeneration commonly associated with Parkinson’s disease. With the increasing problem of population aging, the disease has become a widespread concern in medical and genetic circles. Its symptoms are mainly divided into motor impairments and non-motor impairments. Motor impairments mainly include muscle rigidity, tremor, gait disorder, and bradykinesia, while non-motor impairments such as constipation, gut diseases, anxiety, depression, sleep disorders, and cognitive dysfunction have been found to be earlier than motor impairments in recent years and can seriously affect the quality of life (3). Therefore, our review focuses on the research progress of the gut microbiome and the correlation between Toll-like receptors and Parkinson’s disease. We reviewed the latest evidence that disrupted Toll-like receptor 2 or Toll-like receptor 4 signaling pathway leads to Parkinson’s disease in gut homeostasis and enteric nervous system, as well as gut dysfunction in Parkinson’s disease. We suspect that Toll-like receptor 2 and Toll-like receptor 4 signaling pathways may promote Parkinson’s disease pathogenesis by increasing gut permeability and gut inflammation, thereby driving α-synuclein aggregation in the gut or brain.

## 2 Gut microbiome and Parkinson’s disease

### 2.1 Gut microbiome

The largest microbiome is in the human gut, the gut microbiome, with a number of about 100 trillion and a genome of about 3 million, known as the “second genome” of the human body. These include bacteria, fungi, and viruses, mainly bacteria, accounting for more than 90%. The cultures are interdependent and mutually restrictive, which maintain a symbiotic relationship and a physiological balance with the host. A healthy gut microbiome is essential for maintaining nutrient metabolism and body development, enhancing immunity and anti-aging, and improving tumor immunotherapy (5, 6). The gut microbiome is mainly composed of four bacterial phyla, of which Firmicutes constitute even two-thirds, followed by Bacteroidetes, Actinobacteria, and Proteobacteria (7). Under normal circumstances, the gut microbiome and the host are in a relatively balanced state, but when the gut microbiome is out of balance, it will induce gut dysfunction and then induce a series of diseases.

#### Microbiome–gut–brain axis

The brain can directly or indirectly influence the composition of microorganisms through the release of signaling molecules through the innate cell layers (enteric chromophils, neurons, and immune cells) or by altering gastrointestinal peristalsis function, secretions, and gut permeability. The gut microbiome can also interact with immune regulation pathways, vagus neuromodulation pathways, neuroendocrine regulatory pathways, metabolic system regulatory pathways, and endocrine regulatory pathways by releasing bacterial substrates, metabolites, and gastrointestinal endocrine factors to achieve interaction and communication with the brain, affecting the development and function of the brain and forming a new pathway of two-way communication between “microbiome–gut–brain axis” (8, 9). There is a large amount of lymphoid tissue under the human intestinal mucosa that responds to abnormal changes in the gut microbiome through the immune system, known as gut-associated lymphoid tissues (GALTs), containing approximately 80% of immune components (10). Gut-associated lymphoid tissues include pyle collecting lymph nodes located in the wall of the small gut, which separate lymphoid follicles scattered throughout the gut, appendix, and Wechsler ring. Under normal circumstances, the epithelial tissue of the intestinal mucosa secretes a large amount of mucus, which contains a large amount of mucin, which has the effect of preventing microorganisms from adhering to the epithelium. Germ-free (GF) mice show defects in the gut and systemic immune tissue and increased blood–brain barrier (BBB) permeability, and it is speculated that the gut microbiome may be involved in building the body’s immune system. Germ-free mice also exhibit increased circulating corticosteroid levels (11), suggesting hyperfunction of their hypothalamic–pituitary–adrenal axis. The gut microbiomes are also involved in the production of a variety of neurotransmitters (12), and *Streptococcus*, *Enterococcus*, and *Escherichia* species can produce serotonin. *Lactobacillus* and Bifidobacteria can produce γ-aminobutyric acid. *Escherichia*, Bacilli, and yeast can synthesize norepinephrine. *Enterobacter* secretes dopamine. *Lactobacillus* produces acetylcholine. These neurotransmitters are essential for regulating the body’s mood, cognitive function, and state of consciousness. The gut microbiome can affect the levels of anti-inflammatory and pro-inflammatory factors through immune responses, activate innate and adaptive immunity, and lead to local and systemic inflammatory responses, thereby affecting the central nervous system.

### 2.2 Parkinson’s disease and gut microbiome

Various lines of evidence are converging to implicate chronic gut dysfunction and immune activation as triggers and drivers of Parkinson’s disease pathology. Patients with Parkinson’s disease exhibit a number of features that reflect the diagnosis of inflammatory bowel disease (13), including altered gut microbiome composition and function (14–18), increased gut permeability (19–21), extensive immune activation (22), and extensive gastrointestinal symptoms (e.g., constipation, nausea, and bloating) (23, 24), some of which may appear decades prior to Parkinson’s disease diagnosis (25). Similarly, various genetic (e.g., LRRK2 and TNF-α variants) (26, 27), environmental (e.g., pesticides), or lifestyle factors (diet, exercise, or antibiotic use) (28, 29) increase the risk or progression of Parkinson’s disease and are also associated with gut malformations and inflammatory bowel disease, which itself is considered a risk factor for Parkinson’s disease (30, 31).

#### 2.2.1 Characteristics of the gut microbiome in patients with Parkinson’s disease

Patients with Parkinson’s disease have gut microbiome disturbances that manifest as marked changes in the number of the microbiome. Many researchers have found a significant increase in gut permeability in patients with Parkinson’s disease compared with healthy patients (32–35). Studies have shown that the abundance of Enterobacteriaceae is positively correlated with the severity of Parkinson’s disease (36). Elevated levels of *Escherichia coli* and serum lipopolysaccharide (LPS) in patients with Parkinson’s disease indicate that gut permeability in Parkinson’s disease patients is associated with intestinal endotoxin exposure (37). The gut microbiome also plays a key role in regulating symptoms, with antibiotic therapy improving the expression of α-synuclein in Parkinson’s disease, while oral administration of specific microbial metabolites affects motor impairments, microglial activation, and aggregation of α-synuclein (38). In addition, predictive function analysis highlights the increased expression of genes involved in lipopolysaccharide biosynthesis in fecal samples from Parkinson’s disease patients, which may be associated with peripheral and central inflammation (37). In 2015, Finnish scientist Scheperjans (36) reported that the abundance of Prevotellaceae in the feces of patients with Parkinson’s disease was reduced by 77.6% compared with normal people. The genus participates in the synthesis of mucin in the gut mucosal layer, participates in the degradation of carbohydrates, and produces products such as short-chain fatty acids, vitamin B1, and folic acid (8). At the same time, the above studies also found that the number of *Enterobacter* bacteria in Parkinson’s disease patients with motor impairment is much higher than in Parkinson’s patients with tremors, and it is positively correlated with the severity of symptoms (32). The increase of Enterobacteriaceae bacteria increases serum levels of LPS, which can activate the Toll-like receptor 4 transmembrane signaling pathway and the intracellular nuclear factor κB (NF-κB) and MAPK signaling pathways to cause the body to produce cytoinflammatory factors such as TNF-α, IL-1β, and IL-6, which destroy the BBB and promote the deposition of α-synuclein (39, 40). Parkinson’s disease patients have a significant decrease in the *Clostridium* caseinate in the gut, and butyric acid produced by *Clostridium* caseinate has the function of regulating the gut, inhibiting pathogenic bacteria in the gut, and promoting the growth of beneficial bacteria (36). Many clinical studies have shown dysregulation of the relative abundance of different taxa of the gut microbiome in patients with Parkinson’s disease (**Table 1**). The genetic sequence of the gut microbiome was detected by 16s rDNA high-throughput sequencing, and it was found that the relative abundance of Prevotellaceae (36, 45) decreased in subjects, the relative abundance of Enterobacteriaceae increased, and its expression level was positively correlated with the severity of gait difficulties in 72 Parkinson’s disease patients compared with 72 control subjects (36). In another study, researchers found that elevated levels of *Anaerotruncus* spp., *Clostridium* XIV_a_, and Lachnospiraceae in Parkinson’s disease patients were associated with dyskinesia symptoms in Parkinson’s disease patients, while elevations in *Akkermansia* were associated with non-motor impairments in Parkinson’s disease patients (14). Prevotellaceae and *Akkermansia* are involved in the synthesis and degradation of gut mucin, which may be associated with increased gut permeability in Parkinson’s disease (14, 36). In addition, *Butyricicoccus* and *Clostridium* XIV_b_ were significantly elevated in Parkinson’s disease patients compared with healthy controls, which may be associated with cognitive dysfunction in Parkinson’s disease (41). Studies have proved that the relative abundance of *Blautia*, *Coprococcus*, and *Roseburia* in Parkinson’s disease patients has decreased, among which *Blautia* is considered to be a butyrate-producing bacterium with anti-inflammatory effects, while the relative abundance of *Faecalibacterium* with pro-inflammatory effects is significantly increased (37). Pietrucci et al. (42) found that the presence of lactic acid bacteria (46), Enterobacteriaceae, and *Enterococcus* in the guts of Parkinson’s disease patients was higher than that of ordinary patients, while the number of Lachnospiraceae (42, 47–49) was less. However, Hill-Burns et al. (50) found that the abundance of lactic acid bacteria in the guts of patients with Parkinson’s disease decreased. Experiments have shown that the levels of Bifidobacteriaceae and Rikenellaceae in Parkinson’s disease patients have increased, and the relative abundance of Puniceicoccaceae and *Roseburia* has decreased (45).

**Table 1:** Changes in the gut microbiome in patients with Parkinson’s disease.

Studies	Subjects	Results (H = higher; L = lower)
Scheperjans et al. (36)	72 PD cases and 72 controls	Enterobacteriaceae (H) Prevotellaceae (L)
Heintz-Buschart et al. (14)	76 PD cases and 78 controls	*Anaerotruncus* spp.(H) *Clostridium* XIV_a_ (H) *Akkermansia* (H) Lachnospiraceae (L)
Qian et al. (41)	45 patients and their healthy spouses	*Butyricicoccus* (H) *Clostridium* XIV_b_ (H)
Keshavarzian et al. (37)	38 PD cases and 34 controls	*Faecalibacterium* (H) *Blautia* (L) *Coprococcus* (L) *Roseburia* (L)
Pietrucci et al. (42)	80 PD cases and 72 controls	Lactic acid bacteria (H) Enterobacteriaceae (H) *Enterococcus* (H) Lachnospiraceae (L)
Aho et al. (43)	64 PD cases and 64 controls	Bifidobacteriaceae (H) Rikenellaceae (H) Lachnospiraceae (L) Puniceicoccaceae (L) *Roseburia* (L)
Lin et al. (44)	80 PD cases and 77 controls	*Verrucomicrobia* (H) *Mucispirillum* (H) *Parabacteroides* (H) *Porphyromonas* (H) Lactic acid bacteria (H) Prevotellaceae (L)
Cirstea et al. (45)	197 PD cases and 103 controls	Christensenellaceae (H) Bifidobacteriaceae (H) *Collinsella* (H) *Bilophila* (H) *Akkermansia* (H) Lachnospiraceae (L) *Roseburia* (L) *Faecalibacterium* (L)
Vascellari (46)	64 PD cases and 51 controls	*Akkermansia* (H) *Escherichia* (H) *Bilophila* (H) *Streptococcus* (H) Bacteroidetes (L) *Blautia* (L) Lachnospiraceae (L) *Butyricicoccus* (L) *Roseburia* (L)
Barichella (47)	193 PD cases and 113 controls	Christensenellaceae (H) Lactic acid bacteria (H) *Bilophila* (H) *Parabacteroides* (H) Lachnospiraceae (L) *Roseburia* (L)
Hill-Burns et al. (15)	197 PD cases and 130 controls	Lactic acid bacteria (L) SCFAs (L)

PD, Parkinson’s disease; SCFAs, short-chain fatty acids.

### 2.3 Pathogenesis of Parkinson’s disease

#### 2.3.1 α-Synuclein and Parkinson’s disease

##### 2.3.1.1 α-Synuclein is a pathogenic component of Parkinson’s disease

Gastrointestinal microbial disorders mainly change gut permeability through neuroendocrine and neuroimmune aspects (51, 52), promote the secretion of inflammatory factors and the formation of a pro-inflammatory environment, and induce misfolding and pathological deposition of α-synuclein (53). In Parkinson’s disease, the pathogenic components of Lewy and Lewy neurites are predominantly α-synuclein, which is a native conformationally flexible unfolded protein that exists as monomers and polymers in healthy cells also is involved in the release of neurotransmitters, the transmission of synapses, and the role of mitochondria and lysosomes (54). Many neurodegenerative diseases are attributed to misfolding and consequent aggregation of proteins in the nervous system, leading to neurotoxicity and cell death. Protein misfolding is caused by environmental, genetic, and age factors that affect the physicochemical state of the body, resulting in impaired protein synthesis, folding, and clearance (55). To date, α-synuclein accumulation has been thought to be an abnormal or dysregulated process that causes altered gene expression and degeneration of damaged proteins. This insoluble α-synuclein can be released from neurons in a variety of ways, including exosome-mediated secretion or apoptosis. In the extracellular space, α-synuclein can propagate misfolded proteins or be rapidly absorbed by neighboring cells (56, 57) and transported retrogradely along with neuronal axons (58, 59).

##### 2.3.1.2 α-Synuclein is the immune signaling molecule of Parkinson’s disease

**Figure 1:**
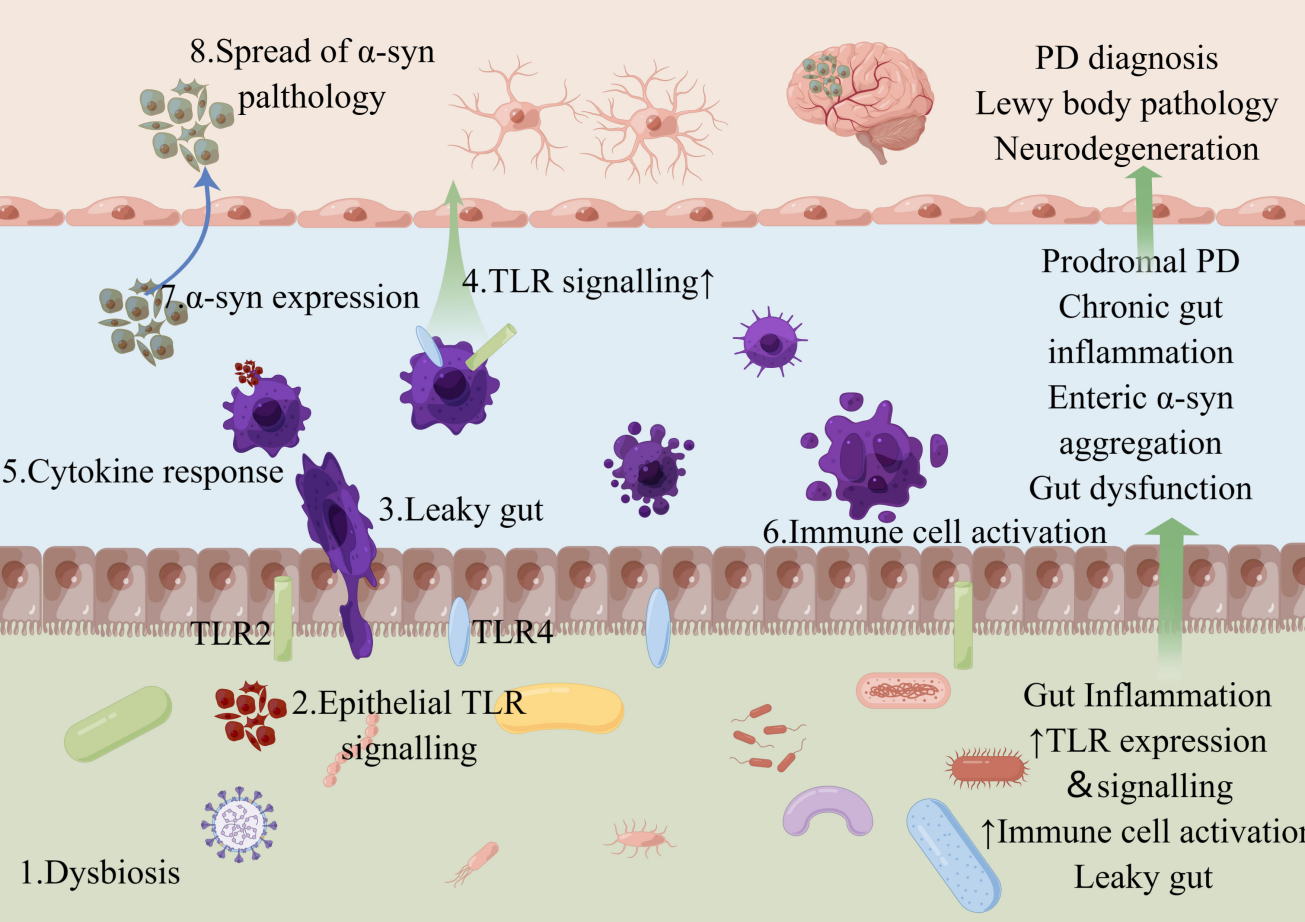
Schematic diagram of the representative distribution of TLR2 and TLR4 in gut. Microbial dysbiosis and precursor gut reactions can lead to complex leaky gut and the notorious positive feedback loop. For example, microbial dysbiosis (1) contributes to the signaling pathway of epithelial TLRs (2), which can result in a leaky gut (3) and further TLR signaling (4) with subsequent cytokine secretion (5) and immune cell activation (6), leading to TLR-associated dysbiosis. Such gut inflammation can alter the enteric nervous system signaling (7). Insoluble α-syn aggregates can spread from the gut to the brain through peripheral nerves, where they cause Lewy body pathology and neurodegeneration of the central nervous system, leading to the characteristic movement disorders of Parkinson’s disease (8). Figure created with Figdraw.com.

Interestingly, emerging evidence suggests that α-synuclein has immune-related functions such that folding, release, and uptake may involve relevant molecular mechanisms rather than pathogenic processes themselves. Therefore, by observing the elevated levels of α-synuclein and the consequent pathology of Parkinson’s disease, we provide an interesting framework for linking the various elements that occur in Parkinson’s disease patients, including risk factors, gut dysfunction, and microbial dysbiosis, and it can be found that gut dysfunction and microbial dysregulation can lead to elevated levels of α-synuclein, which in turn promotes the occurrence and development of Parkinson’s disease. While the role of primary research in synaptic communication in neurons has been conducted, recent studies have shown that α-synuclein functions as an immune signaling molecule (60). Extracellular α-synuclein exerts chemo-attracting effects to stimulate neutrophil and monocytes and dendritic cell maturation *in vitro* (61), as well as α-synuclein as a natural antimicrobial peptide (62, 63) and viral replication inhibitor (51). Thus, α-synuclein is hypothesized to be involved in the mobilization of central and peripheral neurons and enteroendocrine cells in gut epithelial cells (64–66). Abundant α-synuclein is stored in the gut plexus of the mucosa and human appendix (58), and the level of α-synuclein staining of neurons in the gut correlates with the degree of human bowel biopsy (67). Although there are uncertainties regarding the transport, release, and immune-related role of α-synuclein, it is clear that increased expression of α-synuclein elicits pre-inflammatory responses and that changes in the surrounding extracellular microenvironment (e.g., temperature, pH, metal ions, metabolites) can affect the conformation of α-synuclein, leading to protein folding. In turn, the misfolded α-synuclein forms pathological polymers, fibers, and Lewy bodies, which in turn cause lysosomal and mitochondrial dysfunction, interfere with microtubule formation and autophagy, and activate microglia (68, 69). Thus, not only do simple gut dysfunction and resulting exposure to translocation pathogens trigger α-synuclein increases in levels within certain intestinal cells, but long-term intestinal pathological changes may also encourage misfolded α-synuclein aggregation (**Figure 1**), especially when accompanied by overexpression of α-synuclein or disruption of protein clearance (70). In addition, high apoptosis turnover in certain intestinal cell populations means that insoluble α-synuclein aggregates can periodically or chronically be released from enteroendocrine cells into the extracellular space of the gut barrier. α-Synuclein can be secreted in a variety of ways by various cell types, meaning that misfolded α-synuclein in the gut barrier can easily spread into adjacent intestinal neurons, misfolding surrounding proteins and further triggering neurodegeneration. Therefore, intestinal inflammation may increase α-synuclein levels and misfold proteins into a vicious cycle, which may contribute to the development of Parkinson’s disease. Previous gut infections have been associated with the risk of future Parkinson’s disease (71), and pathological Lewy bodies were diagnosed several years ago in patients with gastrointestinal tract extremely pronounced (72). Interestingly, *Helicobacter pylori* infection can worsen motor impairments after diagnosis of Parkinson’s disease (73), suggesting the pathogenesis and progression of infection-mediated gut dysfunction in Parkinson’s disease. In addition, prior viral infections outside the gastrointestinal system may also be associated with the risk of developing Parkinson’s disease, and it is thought that the immune-related effects of α-synuclein lead to elevated protein levels as an endogenous physiological factor, especially in patients with sporadic Parkinson’s disease. As an example, elevated substantia nigra levels in some human immunodeficiency virus (HIV) patients (74) and Western equine encephalitis virus infection cause α-synuclein aggregation with selective dopaminergic loss in mouse brains (75).

#### 2.3.2 Coronavirus disease 2019 and Parkinson’s disease

Of particular note is the two hypothetical starting sites of Parkinson’s disease reflecting in some people the effects of coronavirus disease 2019 (COVID-19) affecting the sense of smell (anosmia) and gastrointestinal tract (microbial dysbiosis, diarrhea, and intestinal inflammation) (76). In addition, there are many similar molecular links between coronavirus disease 2019 and Parkinson’s disease, including oxidative stress, inflammation, and protein aggregation (43). Although there are currently unreliable data linking coronavirus disease 2019 infection or “long-term COVID” to the risk of developing Parkinson’s disease, future studies of the relationship between viral infection, Toll-like receptor signaling pathway, and α-synuclein are critical for Parkinson’s disease. Thus, early gut dysfunction signaling pathways caused by Toll-like receptor 2 and Toll-like receptor 4 destructions may directly lead to elevated α-synuclein levels and aggregation in prodromal Parkinson’s diseases. Furthermore, α-synuclein acts as an agonist of endogenous Toll-like receptor 2 and Toll-like receptor 4 (44, 77), and it is conceivable that elevated Toll-like receptor levels due to pre-existing inflammation or Toll-like receptor initiation can induce a positive feedback loop that can induce α-synuclein–Toll-like receptor signaling pathway. The Toll-like receptor is activated by pathogen-associated molecular patterns and damage-associated molecular patterns, and mediated signaling pathways can regulate the expression of cytokines, chemokines, interferons, and α-synuclein.

## 3 Toll-like receptors

### 3.1 Biological characteristics of Toll-like receptors

Toll-like receptors are a family of receptors closely related to innate immunity, which belong to a family of pattern recognition receptors; widely distributed in human tissues and cells; mainly distributed in the immune system, neurons, and glial cells; and can recognize a variety of pathogen-associated molecular patterns and damage-associated molecular patterns, which are a bridge between innate and adaptive immunity (78) and are able to identify small molecules with highly conserved structures from microorganisms. When bacteria break through the natural physical protective barriers of the human body, such as skin and mucous membranes, Toll-like receptors can recognize and stimulate the human body to form an immune response (79). Medzhitov et al. (80) first discovered in 1997 that the Toll-like receptor of humans homologous to fruit flies can activate the NF-κB signaling pathway, called Toll-like receptors, and at present, a total of 13 Toll-like receptors (i.e., Toll-like receptors 1–13) have been found in the human body, of which Toll-like receptors 3, 7, 8, 9, and 13 are distributed in the nuclear endosomes of cells, and the rest of the Toll-like receptors are distributed on the cell surface (81). These Toll-like receptor family molecules recognize the corresponding ligands, activate downstream signal transduction pathways, trigger immune responses, induce cytokines and chemokines, and play an important role in innate and adaptive immune responses.

#### 3.1.1 Distribution and expression of Toll-like receptor 2 and Toll-like receptor 4

While Toll-like receptor 2 and Toll-like receptor 4 in Toll-like receptors are important sub-receptors for inducing Parkinson’s disease development, Toll-like receptor 2 and Toll-like receptor 4 are generally expressed in a healthy gut but are more obvious in the colon. When the gut reacts, Toll-like receptor 2 and Toll-like receptor 4 are activated by recognizing bacterial DNA in the gut, the expression of Toll-like receptor 2 and Toll-like receptor 4 in the gut is significantly higher than in healthy people (82), and abnormal expression of their receptors can accelerate the occurrence of Parkinson’s disease (83). Toll-like receptors located on the cell surface recognize multiple components of bacteria and viruses, including LPS, peptidoglycan, flagellin, and lipoprotein. Toll-like receptors located on endosomal membranes recognize nucleic acids of bacteria and viruses. Toll-like receptors mediate the specific recognition of exogenous pathogen-associated molecular patterns, which are secreted by bacteria, viruses, fungi, parasites, etc., and endogenous injury-associated molecular patterns, which are secreted by damaged tissues or cells. For example, Toll-like receptor 2 mainly acts on macrophages, monocytes, and epithelial cells of part of the mucosa of the digestive tract, etc., and plays a role by recognizing gram-positive bacterial components (84). Toll-like receptor 4 is highly expressed in human renal tubular epithelial cells and intestinal epithelial cells, and Toll-like receptor 4 is mainly effective for the recognition of LPS of gram-negative bacteria and is the only receptor for LPS (85, 86). It activates the transduction pathway of the cross-membrane immune signaling pathway mediated by Toll-like receptor 2 and Toll-like receptor 4, thereby mediating the inflammatory response, initiating the immune system, and producing inflammatory factors (87–89).

### 3.2 Toll-like receptor 4

To reflect the inflammatory nature of Parkinson’s disease, a growing body of research supports altered Toll-like receptor 4 signaling pathway in Parkinson’s disease pathology. Patients with Parkinson’s disease exhibit elevated levels of Toll-like receptor 4 protein and putamen samples in colon biopsies, circulating monocytes, and postmortem caudate nucleates (90). Studies have also shown that deranged serum markers indicate Toll-like receptor 4 activation (as assessed by levels of altered LPS-binding proteins) (19). Animal experiments have found that intraperitoneal injection of 1-methyl-4-phenyl-1,2,3,6-tetrahydropyridine (MPTP) into mice with MPTP showed less microglial activation and less dopamine neuronal loss in the substantia–striatum nigra site of Toll-like receptor 4-deficient mice compared with wild-type mice (91). Cell experiments have also confirmed that Toll-like receptor 4 expression is upregulated in 1-methyl-4-phenylpyridinium (MPP^+^)-induced BV-2 cells (92). Autopsy of Parkinson’s disease patients has seen an increased expression of Toll-like receptor 4 in the substantia nigra of the midbrain (93, 94). The above studies strongly suggest that Toll-like receptor 4 plays an important role in the pathogenesis and progression of Parkinson’s disease.

#### 3.2.1 Structural function of Toll-like receptor 4

Toll-like receptor 4 is a pattern recognition receptor that belongs to type I transmembrane glycoprotein, which is mainly expressed in innate immune cells such as macrophages and dendritic cells and mainly exists in the brain in microglia, astrocytes, and other cells involved in the immune response of the central nervous system. Toll-like receptor 4 is activated by recognizing the LPS of gram-negative bacteria, the F protein of the respiratory syncytial virus, glycerol inositol phospholipids of trypanosomes, and damage-associated molecular patterns such as high mobility group protein B1 (HMGB1) and heat shock proteins (HSPs) (95). The main structures and functions of Toll-like receptor 4 are as follows: 1) leucine-rich extracellular domain: recognizes the pathogen-associated molecular pattern and damage-associated molecular pattern to form receptor complexes; 2) intracellular Toll/interleukin-1 (IL-1) receptor (TIR) domain: homologous to the IL-1 receptor, responsible for recruiting downstream adaptor factors, triggering signal transduction from intracellular to nucleus. After Toll-like receptor 4 activation, oligomerization occurs and then the activation of myeloid differentiation factor 88 (MyD88), TIR domain-containing adaptor protein inducing interferon-β (TRIF), and TRIF-associated adaptor. In TRIF-related adaptor molecule (TRAM) and other downstream adaptor factors, a series of cascade biochemical reactions occur after the adaptor protein is activated, initiating downstream signaling pathway and finally transmitting the signal from the cell to the nucleus, subsequently activating NF-κB, activator protein-1 (AP-1), signal transduction and signal transducer and activator of transcription 3 (STAT3), and other transcription factors. Different transcription factors initiate the transcription and protein expression of corresponding target genes, such as inducing the production and release of tumor necrosis factor-α (TNF-α), IL-1β, IL-6, and other pro-inflammatory cytokines, and exerting anti-infection, autoimmune, and other effects. After Toll-like receptor 4 is activated, on the one hand, it initiates the innate immune response, which is equivalent to the “electric gate” of innate immunity. On the other hand, adaptive immune responses can also be regulated by releasing chemokines and regulating T-cell differentiation (80).

#### 3.2.2 Toll-like receptor 4 signal transduction

The Toll-like receptor 4 signaling pathway is complex and involves the participation of a variety of molecules and linker factors. The pathological mechanisms of various diseases such as chronic gastrointestinal diseases and malignant tumors are related to abnormal activation of Toll-like receptor 4, and all suggest that the transduction mode of the Toll-like receptor 4 signaling pathway is complex and fine. Parkinson’s disease is no exception, and the Toll-like receptor 4 signaling pathway involves multiple pathways in pathogenesis studies, starting with the classical Toll-like receptor 4/NF-κB signaling pathway, which is involved in the immune inflammatory response (96, 97). Toll-like receptor 4 deficiency has been found to significantly attenuate the activation of the Toll-like receptor 4/NF-κB signaling pathway in the midbrain of MPTP-induced Parkinson’s disease mice and reduce neuroinflammation (98, 99). In addition, in the pathological process of Parkinson’s disease, Toll-like receptor 4 can also be associated with mitogen-activated protein kinases (MAPKs) that regulate cell growth and differentiation, glycogen synthase kinase 3β (GSK3β), AP-1 that regulates oxidative stress and nuclear factor E2 (Nrf2), and other factors that form a cascading pathway. Paraquat-activated BV2 cells have shown that Toll-like receptor 4 inhibitors significantly reverse the phosphorylation of MAPKs and reduce transcription levels of pro-inflammatory cytokines compared with MAPK inhibitors (100). Studies in animal models of Parkinson’s disease have also found that activation of the Toll-like receptor 4/TNF receptor-associated factor 6 (TRAF6)-mediated MAPK signaling pathway is involved in neuroinflammatory processes (96). Similarly, in LPS-induced models of cellular inflammation, the Toll-like receptor 4/MAPK signaling pathway has been found to be involved in activating microglia, ultimately causing neuronal inflammatory damage induced by SHSY5Y cells (97). In addition, Shao et al. (101) found in the study of α-synuclein-stimulated neuroinflammation that TNF-α expression is regulated by the Toll-like receptor 4/phosphatidylinositol 3-kinase (PI3K)/AKT/GSK3β signaling pathway. There have also been studies showing that MPTP-induced Toll-like receptor 4−/− mice exhibit downregulation of AP-1 expression (99). In LPS-induced astrocyte models, Toll-like receptor 4 activation causes downregulation of Nrf2 expression (102). The limitation of this research is that there is no research to prove the possible intricate relationship between the above individual pathways, such as series, parallel, or cascade, and there is an urgent need for large-sample experiments and human trials with clear ideas to better elucidate the signal transduction and mechanism of action of Toll-like receptor 4 in Parkinson’s disease.

#### 3.2.3 Toll-like receptor 4 and α-synuclein

In recent years, the relationship between α-synuclein and Toll-like receptor 4 has been controversial. Stefanova et al. (103) found that both the functional blockade and gene knockout of Toll-like receptor 4 could lead to a decrease in the phagocytosis of α-synuclein by microglia, resulting in the accumulation of α-synuclein in the mouse brain and the decrease in dopamine neurons. A similar study (51) also confirmed increased aggregation of α-synuclein protein in the midbrain of Toll-like receptor 4−/− mice and increased expression of α-synuclein mRNA in the cerebral cortex, striatum, hippocampus, and cerebellum. Based on the fact that α-synuclein aggregation first appeared in the olfactory bulb and vagus nerve (104), Chen et al. (105) conducted a study on the pathological tissue of the olfactory bulb and found that the Toll-like receptor/NF-κB signaling pathway was activated in the olfactory bulb of Parkinson’s disease model mice and the phosphorylation level of p65 protein was significantly increased. Based on the above studies, microglia may regulate the synthesis, clearance, and distribution of α-synuclein in the brain in a Toll-like receptor 4-dependent manner, and Toll-like receptor 4 signaling pathway disorders or signal transduction disorders can cause α-synuclein aggregation and lead to Parkinson’s disease.

Studies have found that α-synuclein can be used as a Toll-like receptor 4 activator to induce activation of the Toll-like receptor 4 signaling pathway, causing neuroinflammation dominated by TNF-α high expression (101). When Toll-like receptor 4−/− macrophages are stimulated by α-synuclein oligomers, pro-inflammatory factor secretion is significantly reduced (94). Increasing the dose of α-synuclein oligomers at physiological concentrations sensitizes Toll-like receptor 4 on the surface of microglia and astrocytes, increasing their downstream production of pro-inflammatory cytokines (94). In addition, *ex vivo* studies of astrocytes have found that recombinant human α-synuclein and oligomers can activate the Toll-like receptor 4 signaling pathway in *ex vivo* primary astrocytes, leading to pro-inflammatory factor production, but the uptake of α-synuclein by astrocytes is not associated with Toll-like receptor 4 (94, 106). It has also been confirmed that α-synuclein induces C-X-C mode-ordered chemokine ligand 12 by activating the Toll-like receptor 4 signaling pathway (C-X-C motif chemokine ligand 12, CXCL12; a classical inflammatory chemokine), and CXCL12 is involved in α-synuclein-induced microglial accumulation (107). It can be seen that α-synuclein seems to activate Toll-like receptor 4 in this “suicidal” way, thereby mediating its clearance of itself. Of course, the inflammatory pathological process that relies on α-synuclein Toll-like receptor 4 activations may also be an early cause of Parkinson’s disease.

#### 3.2.4 Toll-like receptor 4: the bridge between coronavirus disease 2019 and Parkinson’s disease

Although the exact mechanism of Parkinson’s disease pathogenesis is unknown, in the activation of Toll-like receptor, primarily Toll-like receptor 4 and subsequent neuroinflammation, the immune response appears to play an important role. There is growing evidence suggesting that viral infections may be consistent with the precipitation of Parkinson’s disease or Parkinsonism. The recently identified coronavirus, named severe acute respiratory syndrome coronavirus 2 (SARS-CoV-2), is the causative agent of the ongoing pandemic coronavirus disease 2019, causing 160 million cases and more than 3 million deaths worldwide. The binding of the SARS-CoV-2 spike (S) protein to Toll-like receptor 4 and the possible interaction between SARS-CoV-2 and α-synuclein were also considered contributing factors to neuronal death. High levels of ACE2 and TMPRSS2 in neurons, astrocytes, and oligodendrocytes may promote a neuroinvasive predisposition to SARS-CoV-2, and these levels, together with Toll-like receptor 4 activation, predispose patients to α-synuclein aggregation, neurodegeneration, and Parkinson’s disease pathogenesis (108).

##### 3.2.4.1 α-Synuclein between coronavirus disease 2019 and Parkinson’s disease

At present, the possible impact of coronavirus disease 2019 on the pathogenesis of Parkinson’s disease is controversial. Parkinson’s disease is associated with central nervous system immune response, microglial and oligodendrocytes activation, histocompatibility class II upregulation, and pro-inflammatory cytokine overproduction. Severe acute respiratory syndrome coronavirus 2 infection can be a substantial immune response. Cytokine storms during coronavirus disease 2019 can lead to blood–brain barrier breakdown and lead to viral entry and immune cell infiltration (109). This can directly lead to neuronal death and escalation of patient care to severe neurological complications that characterize all synucleinopathies, including Parkinson’s disease (110–113). Parkinson’s syndrome has been reported following coronavirus disease 2019. Although Parkinson’s disease was not diagnosed, functional nigrostriatal neuroimaging was abnormal in some coronavirus disease 2019 cases, therefore presuming dopaminergic nigrostriatal impairment (114, 115). Moreover, some of the most common non-motor impairments of Parkinson’s disease are evident in coronavirus disease 2019 patients and comprise anosmia/hyposmia, gastrointestinal symptoms, ageusia, fatigue, and painful limbs (116, 117). These symptoms antedate the pathological deposition of α-synuclein and the appearance of motor impairments. α-Synuclein is the most important protein implicated in Parkinson’s disease. Similar to West Nile virus and severe acute respiratory syndrome coronavirus 1, severe acute respiratory syndrome coronavirus 2 infection may cause α-synuclein upregulation in an attempt to prevent viral replication and nerve invasion (61, 118). However, abundant and persistent systemic inflammation may disrupt host cell protein balance and protein quality control systems, leading to pathological modification of α-synuclein, which can develop with the formation of fibril structures. Despite the need for confirmation, recent findings suggest that interactions between severe acute respiratory syndrome coronavirus 2 N proteins and α-synuclein can accelerate protein aggregation into amyloid fibers, leading to reproduction and extensive neurodegeneration (119). In addition, α-synuclein aggregation may also result from interactions between severe acute respiratory syndrome coronavirus 2 and damaged proteins that belong to the autophagy mechanism or are involved in maintaining protein balance.

### 3.3 Toll-like receptor 2

Toll-like receptor 2 is a cell surface receptor expressed in various immune cells and epithelial cells, as well as cells in the central, peripheral, and enteric nervous systems of neurons and glial cells (120). Toll-like receptor 2 forms homodimeric or heterodimer Toll-like receptor 1 and Toll-like receptor 6 to recognize a variety of gram-negative and gram-positive bacterial products such as lipophosphate, lipoprotein, peptidoglycan, and bacterial amyloid (e.g., Curli protein) (121), as well as endogenous substances including α-synuclein. Activation of Toll-like receptor 2 may lead to pre-inflammatory as well as anti-inflammatory responses depending on cell type and ligands and co-receptors (122). For example, on dendritic cells, activation of Toll-like receptor 2 by peptidoglycan (rather than lipoteichoic acid) induces TNF-α release. Interestingly, activation of Toll-like receptor 2 through the accumulation of Aβ42 aggregates in microglia elicits protocytokine release, a response enhanced by Toll-like receptor 1 coreceptor action but inhibited Toll-like receptor 2-Toll-like receptor 6 activation (122). Conversely, activation of Toll-like receptor 2 by polysaccharide A from *Bacteroides fragilis* causes an anti-inflammatory response in B cells resulting in interleukin-10 (IL-10) production (123), suggesting that Toll-like receptor 2 has complex immunomodulatory effects in the gut or endogenous factors, which are associated with neurodegeneration.

#### 3.3.1 Expression of Toll-like receptor 2 in Parkinson’s disease patients

Given its established role in innate immunity, Toll-like receptor 2 may play a key role in microbiome-induced systemic action, and there are several pieces of evidence that the Toll-like receptor 2 signaling pathway is altered in Parkinson’s disease patients. First, polymorphisms in C–T single-nucleotide Toll-like receptor 2 rs3804099 are associated with an increased risk of Parkinson’s disease in Han populations, especially in patients with late-onset Parkinson’s disease (124), while the recent use of the Caucasus subset for research progress markers (PPMI) cohort reported that TC heterozygotes and minor CC homozygous Toll-like receptor 2 rs3804099 significantly increased the risk of Parkinson’s disease (125), indicating that possible regional or ethnic differences play a role in the polymorphism of Toll-like receptor 2 and Parkinson’s disease. This polymorphism is an important expression quantitative trait locus (eQTL) in some cell types, is expected to affect Toll-like receptor 2 mRNA levels (126), and is associated with bacterial susceptibility to infection (126, 127), but further studies are needed to confirm the direct effects of Toll-like receptor 2 rs3804099 on protein levels.

Toll-like receptor 2 expression is increased in monocytes in Parkinson’s disease patients (93, 128). Brain samples from Parkinson’s disease patients analyzed after the fact have demonstrated increased Toll-like receptor 2 expression in neurons and microglia in various regions of the brain, including the substantia nigra and putamen (129), as well as increased expression of the substantia nigra striatum Toll-like receptor 2-related signaling pathways [including cluster 14 (CD14), a coreceptor for Toll-like receptor 4] (130). In healthy brains after death, Toll-like receptor 2 levels are particularly predominantly low in microglia (94). Thus, it has consistently been suggested that elevated neuronal Toll-like receptor 2 levels are a consequence of the disease process in Parkinson’s disease patients (131). Interestingly, Toll-like receptor 2 expression in the substantia nigra of Parkinson’s disease patients was significantly higher than in healthy controls but significantly lower than in sporadic Lewy body disease (132) (prodromal phase of Parkinson’s disease and asymptomatic phase), thus supporting Toll-like receptor 2 expression in the early disease process. In addition, this study reports an association between Toll-like receptor 2 expression in the substantia nigra striatum and occasional microglia initiated in Lewy body disease, leading the authors to suggest that upregulation of Toll-like receptor 2 may represent widespread Parkinson’s disease pathology and neurodegeneration and that this early microglial activation may be due to interactions between Toll-like receptor 2 and oligomeric α-synuclein (132).

#### 3.3.2 Toll-like receptor 2 and α-synuclein

Studies have also shown a functional molecular link between histopathology and Toll-like receptor 2 and α-synuclein. Notably, Toll-like receptor 2 is colocalized with α-synuclein in the anterior cingulate cortex of postmortem brain tissue in Parkinson’s disease patients (129). At the cellular level, α-synuclein is an endogenous Toll-like receptor 2 agonist and directly causes microglial activation and upregulation of Toll-like receptor 2 expression (132). Toll-like receptor 2 activation increases the uptake of α-synuclein fibrils by neurons, astrocytes, and microglia (129), with different potential mechanisms and consequences (133). In neurons and astrocytes, Toll-like receptor 2 stimulation impairs α-synuclein fibril degradation (133), and treatment of SH-SY5Y and primary human neurons using the Toll-like receptor 2 agonist PAM3CSK4 culture (but not the Toll-like receptor 4 agonist LPS) increases α-synuclein levels due to lysosomal degradation or impaired autophagy (129). Correspondingly, Toll-like receptor 2 activation inhibits autophagy by activating AKT/mTOR, while inhibition of Toll-like receptor 2 signaling pathway reduces neuronal α-synuclein overexpression in Toll-like receptor 2 knockout mice (134). Thus, Toll-like receptor 2 activation in neurons and astrocytes increases α-synuclein uptake and impairs degradation processes, leading to neurotoxicity. In contrast, the α-synuclein released by the cells activates microglia, in which α-synuclein Toll-like receptor 2 stimulates a pre-inflammatory response and α-synuclein internalization (130) but is degraded in a Toll-like receptor 2-independent manner (133). Cultures expose microglia to recombinant α-synuclein-activated cells and increase Toll-like receptor 2 expression (135). In addition to cell type differences in α-synuclein Toll-like receptor 2 responses, conformational α-synuclein also plays a role. Studies in primary rat microglia cultures and Toll-like receptor 2 knockout mice have shown that oligomeric α-synuclein secreted in a Toll-like receptor 2-dependent manner from neurons (130) causes an inflammatory response by Toll-like receptor 2 signal transduction in human monocytes by fibril α-synuclein aggregates (but not oligomers) in human monocytes (136), indicating cellular and conformational responses to α-synuclein. Similarly, a recent paper exposing microglia to different α-synuclein conformations *in vitro* showed that α-synuclein monomers and oligomers activate NLRP3 via Toll-like receptor 2 and Toll-like receptor 5. While fibrils cause NLRP3 activation, microglial inflammasomes are not activated after initial initiation with LPS and “band” conformation, of which NLRP3 inflammasomes are important for α-synuclein phagocytosis and decomposition (137). Toll-like receptor 2 may be an effective therapeutic target for Parkinson’s disease because blocking Toll-like receptor 2 with functional inhibitory antibodies reduces the transmission of α-synuclein and the accumulation of α-synuclein between neurons and astrocytes between cells and improves nerve damage and neurodegenerative disease in mice with behavioral disorders of α-synuclein overexpression (138). Further research is needed to investigate the role of Toll-like receptor 2 with α-synuclein gut–brain transmission and the role of Toll-like receptor 2 and α-synuclein in immune cells, intestinal neurons, and intestinal cells such as enteroendocrine cells.

#### 3.3.3 Toll-like receptor 2 and Toll-like receptor 4 interact with each other in the gut microbiome

Gut Toll-like receptor has wide-ranging effects on intestinal physiology, regulatory dynamics, epithelial barrier integrity, mucosal immune response (139), and enteric nervous system function.

As mentioned earlier, the gut lumen houses the gut microbiome, which co-evolved with humans. The gut microbiome has been a hot topic for the past two decades and has implications for scientific endeavors, and it is clear that the gut microbiome connects all aspects of health, communication, and influence on human biology (e.g., the microbiome–immune axis, the gut–brain axis, the gut–skin axis). Of note, the effects of antibiotics on the gut microbiome on development and overall health were determined using sterile animals born under sterile conditions without a gut microbiome or microbiome depletion model. For example, in the GF rodent model, immune development, stress response capacity, learning capacity, memory and affective capacity, and social behavior are altered (140–142). Interestingly, a family model of Parkinson’s disease in Thy1-α-synuclein overexpressed mice, reared under GF conditions, showed that mice with α-synuclein-dependent microglia had less locomotor and gastrointestinal tract than mice cloned with complex microbiome (38). Similarly, in rodents, oral antibiotics improved 6-hydroxydopamine (6-OHDA; another established Parkinson’s disease model), suggesting that microbiome-driven signal transduction exacerbates central neurodegeneration of Parkinson’s disease (143). While these conclusions highlight the role of the microbiome and microbiome-driven inflammation in Parkinson’s disease, as well as the complexity of the disease, further clinical studies are needed to understand how the composition of the microbiome relates to changes in humans.Microbial dysbiosis is widely recognized as evident in patients with Parkinson’s disease (14–18). However, the complexity of dynamic microbiomes, the complexity of microbiome–host interactions, and the heterogeneity of Parkinson’s disease complicate the complexity of knowing which microbiome change directly helps to understand the pathogenesis of Parkinson’s disease, which is the result of an ongoing disease process or disease-related lifestyle changes, or may simply reflect an individual’s unique microbial “fingerprint”. Moreover, not everyone with microbiome dysbiosis will develop Parkinson’s disease. This complexity only underscores the need for investigation, which may mitigate dysbiosis as well as the decline of pre-inflammatory responses. In fact, the Toll-like receptor has been reviewed earlier as a mediator of the microbiome–gut–brain axis and innate immune system in Parkinson’s disease patients (79), and this section focuses on the role of Toll-like receptor 2 and Toll-like receptor 4 in overall intestinal physiology and the possible role of local effects on early Parkinson’s disease pathogenesis and enteric nervous system function.

In the gut, Toll-like receptor, especially Toll-like receptor 2 and Toll-like receptor 4, is an integral part of normal intestinal homeostasis, improving microbial tolerance, improving gut barrier integrity, and regulating immune responses to pathogens. A variety of environmental, genetic, and microbial factors can influence the Toll-like receptor signaling pathway, and long-term exposure to pro-inflammatory factors (e.g., diet, infectious agents, and antibiotics) can lead to chronic inflammation, microbial dysbiosis, and leaky gut. This gut dysfunction, especially the translocation barrier of pathogenic microorganisms in leaking gut, can trigger α-synuclein release from certain intestinal cell populations as part of a normal immune response. According to Braak’s hypothesis (144), α-synuclein aggregates have been reported to travel from the gut to the brain via the vagus nerve, where α-synuclein aggregates ultimately trigger Toll-like receptor 2- and Toll-like receptor 4-mediated inflammation and microglial activation leading to neurodegenerative and motion injury in Parkinson’s disease patients. Therefore, it is conceivable that chronic Toll-like receptor-mediated gut dysfunction and intestinal inflammation may lead to the α-synuclein propagating through the entire gut and eventually to the brain. Alternatively, α-synuclein that accumulates locally in the gut may contribute to chronic inflammatory circulation and dysfunction, ultimately triggering systemic inflammation. In turn, the systemic inflammatory response is predominantly central immune receptors (including Toll-like receptor 2/4), which can alter the permeability of the blood–brain barrier leading to central nerve damage, which is directly thought to trigger central α-synuclein aggregation and neurodegeneration of the substantia nigra. Further research into the role of α-synuclein in the gut requires validating these hypotheses and understanding whether and how normal immune mechanisms become pathogenic, leading to Parkinson’s disease.

## 4 Outlook

In conclusion, according to the existing theoretical studies and experiments, the intestinal microbial and enterogenic inflammatory response plays an important role in the inflammatory response of the central nervous system and the pathogenesis of Parkinson’s disease, and the Toll-like receptor signaling pathways play a regulatory role in this process. However, the current research on Toll-like receptor signaling pathways in Parkinson’s disease is not perfect, and further research is still needed on the relationship between Toll-like receptor, the gut microbiome, and Parkinson’s disease. By silencing and knocking out Toll-like receptor-related genes or transferring Toll-like receptor genes into animal models with Toll-like receptor gene defects, animal models of intestinal microbial disorders and α-synuclein aggregation in the brain were observed, and the expression of Toll-like receptor in the substantia nigra and anterior cingulate gyrus cortex was specifically detected so as to explore the relationship between the three. In addition, further studies should be conducted on the amount of inflammatory factor release caused by Toll-like receptors and changes in T lymphocyte function, and the specific signaling pathways of Toll-like receptor-induced intestinal inflammatory response should be analyzed.

Parkinson’s disease is a complex, multifaceted disease with enormous clinical and biological heterogeneity. Studies on gut dysfunction in Parkinson’s disease progression continue to increase, and there is growing evidence that intestinal malformations may contribute to the onset of at least some Parkinson’s disease, although the exact mechanism is unclear. Here, we discuss the role of Toll-like receptor 2 and Toll-like receptor 4 in normal gut functions and provide evidence of disruption of Toll-like receptor 2 and Toll-like receptor 4 signaling pathway, by inherent biological or external factors, that may lead to early gut dysfunction and the evolution of neurodegenerative processes. However, few identified genetic and environmental risk factors for Parkinson’s disease predict risk 100%, and Toll-like receptor 2 and Toll-like receptor 4 expression, function, and signaling pathway are caused by a variety of genetic and environmental factors, many of which are implicated in Parkinson’s disease. Therefore, Toll-like receptor 2 and Toll-like receptor 4 are likely to affect candidates for leaky gut and inflammatory responses, intestinal denervation, and early colonic motility disorders, all of which may help explore the pathogenesis of Parkinson’s disease. However, targeting Toll-like receptor 2 and Toll-like receptor 4 may have diagnostic potential to predict patients at risk of Parkinson’s disease or to stratify disease progression. Currently, most Parkinson’s disease therapies target the brain to improve symptoms of dopamine loss, thus doing little to prevent further neurodegenerative or non-motor impairments. However, targeting Toll-like receptor 2 and Toll-like receptor 4 in the gut may exert multiple benefits when administered alone or in combination with current Parkinson’s disease therapies. Beneficial effects may include relieving microbial dysbiosis and intestinal inflammation to improve gastrointestinal symptoms; improving metabolism of oral Parkinson’s disease drugs and their absorption in the gut, thereby reducing the side effects of increasing doses; and improving systemic inflammation, which can be beneficial to the central nervous system. Similarly, considering Parkinson’s disease pathology, Toll-like receptor 2/4 in the gut is targeted to mitigate α-synuclein aggregation and pathological changes by modifying the gut–brain axis diffusion pattern through lifestyle interventions, probiotics, or specific therapies. Therefore, Toll-like receptor 2 and Toll-like receptor 4 are potential targets to develop new therapies to alter the course of disease in people at high risk of Parkinson’s disease and improve gut disorders in patients in the prodromal phase or with established disease. However, the utility of this treatment is currently limited, especially given the complex role of extensive and Toll-like receptor signaling throughout the body and the heterogeneity of Parkinson’s disease. Therefore, further investigation of Toll-like receptor 2/4-mediated gut dysfunction for Parkinson’s pathogenesis and pathology is urgently needed.

## Author contributions

ZZ wrote and reviewed the manuscript. ZL, AL and CF reviewed and proofread the manuscript. All four authors provided substantial contributions to this review, drafted and critically revised the manuscript, and designed and created the figures. All authors contributed to the article and approved the submitted version.
